# Overlapping Projections of Neighboring Direct and Indirect Pathway Neostriatal Neurons to Globus Pallidus External Segment

**DOI:** 10.1016/j.isci.2020.101409

**Published:** 2020-09-01

**Authors:** Shinichiro Okamoto, Jaerin Sohn, Takuma Tanaka, Megumu Takahashi, Yoko Ishida, Kenta Yamauchi, Masato Koike, Fumino Fujiyama, Hiroyuki Hioki

**Affiliations:** 1 Department of Cell Biology and Neuroscience, Juntendo University Graduate School of Medicine, 2-1-1 Hongo, Bunkyo-ku, Tokyo 113-8421, Japan; 2 Advanced Research Institute for Health Sciences, Juntendo University, 2-1-1 Hongo, Bunkyo-ku, Tokyo 113-8421, Japan; 3 Department of Neuroscience, Graduate School of Medicine, Kyoto University, Yoshida-Konoe-cho, Sakyo-ku, Kyoto 606-8501, Japan; 4 Division of Cerebral Circuitry, National Institute for Physiological Sciences, 5-1 Higashiyama Myodaiji, Okazaki, Aichi 444-8787, Japan; 5 Graduate School of Data Science, Shiga University, 1-1-1 Banba, Hikone, Shiga 522-8522, Japan; 6 Laboratory of Neural Circuitry, Graduate School of Brain Science, Doshisha University, 1-3 Tatara Miyakodani, Kyotanabe, Kyoto 610-0394, Japan

**Keywords:** Biological Sciences, Neuroscience, Cellular Neuroscience

## Abstract

Indirect pathway medium-sized spiny neurons (iMSNs) in the neostriatum are well known to project to the external segment of the globus pallidus (GPe). Although direct MSNs (dMSNs) also send axon collaterals to the GPe, it remains unclear how dMSNs and iMSNs converge within the GPe. Here, we selectively labeled neighboring dMSNs and iMSNs with green and red fluorescent proteins using an adeno-associated virus vector and examined axonal projections of dMSNs and iMSNs to the GPe in mice. Both dMSNs and iMSNs formed two axonal arborizations displaying topographical projections in the dorsoventral and mediolateral planes. iMSNs displayed a wider and denser axon distribution, which included that of dMSNs. Density peaks of dMSN and iMSN axons almost overlapped, revealing convergence of dMSN axons in the center of iMSN projection fields. These overlapping projections suggest that dMSNs and iMSNs may work cooperatively via interactions within the GPe.

## Highlights

• Neighboring dMSNs and iMSNs were exclusively labeled with GFP and RFP
• dMSNs and iMSNs formed two axonal arborizations in the GPe in a topographic manner
• Axonal arborizations of dMSNs were embedded within the arborizations of iMSNs
• The centers of projections from neighboring dMSNs and iMSNs were almost overlapped

## Graphical Abstract

**Figure:**
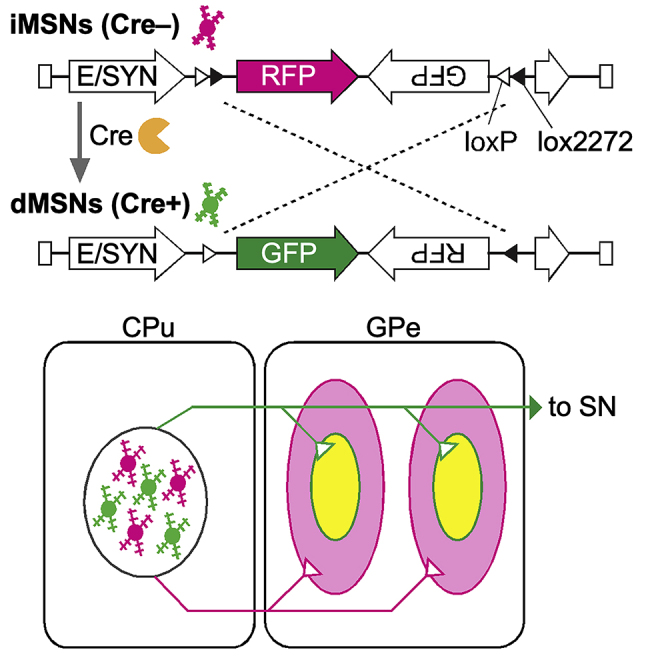

## Introduction

The neostriatum, caudate-putamen (CPu), is the major input nucleus of the basal ganglia, which plays a key role in motor control (Albin et al., 1989; DeLong, 1990) and receives excitatory inputs mainly from the cerebral cortex and thalamus (Alexander and Crutcher, 1990). Medium-sized spiny neurons (MSNs), projection neurons in the CPu, send information to the external segment of the globus pallidus (GPe), entopeduncular nucleus (EP; rodent homolog of the internal segment of the primate globus pallidus), and substantia nigra pars reticulata (SNr), the latter two structures being output nuclei of the basal ganglia (Alexander and Crutcher, 1990). Unraveling the characteristics of these neurons and their axonal distributions that relay information from the upstream to the downstream nuclei is crucial for understanding the function of the basal ganglia.

Projections from the CPu can be divided into two distinct pathways: direct pathway neurons (dMSNs) target the EP and SNr directly, whereas indirect pathway neurons (iMSNs) project to the GPe, and GPe neurons then send axons to the output nuclei via the subthalamic nucleus (Alexander and Crutcher, 1990). In the traditional model of the basal ganglia, direct and indirect pathways have been considered to play opposing roles: the direct pathway delivers a “go signal” for action promotion, whereas the indirect pathway sends a “no-go signal” to suppress movement (Albin et al., 1989; DeLong, 1990). However, accumulating evidence supports coordinated interactions between the direct and indirect pathways (Peak et al., 2019; Tecuapetla et al., 2014): for example, dMSNs and iMSNs can be cooperatively activated during motor action initiation (Cui et al., 2013). These findings indicate that the direct and indirect pathways are not always competing but that a structural basis for the coordinated functions of dMSNs and iMSNs may underlie motor control by the basal ganglia.

dMSNs and iMSNs mainly project to different nuclei in the basal ganglia, but it has been reported that their outputs converge in the GPe. Although dMSNs project predominantly to the EP and SNr, several studies have revealed that dMSNs have axon collaterals in the GPe (Fujiyama et al., 2011; Kawaguchi et al., 1990; Wu et al., 2000). Indeed, an electrophysiological study has revealed that both iMSNs and dMSNs inhibit the activity of GPe neurons (Cazorla et al., 2014). Although the inhibitory effect of both dMSNs and iMSNs on GPe neuron activity may partially contribute to their coordinated functions, the individual projection patterns of dMSNs and iMSNs remain to be clarified. Therefore, to understand how dMSNs and iMSNs cooperatively play a role in motor control, it might be essential to anatomically clarify the convergence of dMSN and iMSN axons in the GPe and identify the corresponding neural circuitry in the basal ganglia.

In the present study, we explored the precise distributions of the varicose fibers of dMSNs and iMSNs in the mouse GPe. We injected an adeno-associated virus (AAV) vector into the CPu of dopamine receptor D1 (Drd1)-Cre transgenic mice (Gong et al., 2007) and labeled dMSNs and iMSNs with green (GFP) and red fluorescent proteins (RFP), respectively. In addition, the palmitoylation signal sequence attached to GFP and RFP enables efficient visualization of axon fibers (Furuta et al., 2001; Kameda et al., 2008; Nishino et al., 2008). We quantitatively analyzed the convergence of dMSN and iMSN axon fibers in the GPe and found a unique distribution of dMSN axons in relation to the iMSN projections.

## Results

### Selective Labeling of dMSNs and iMSNs

**Figure 1:**
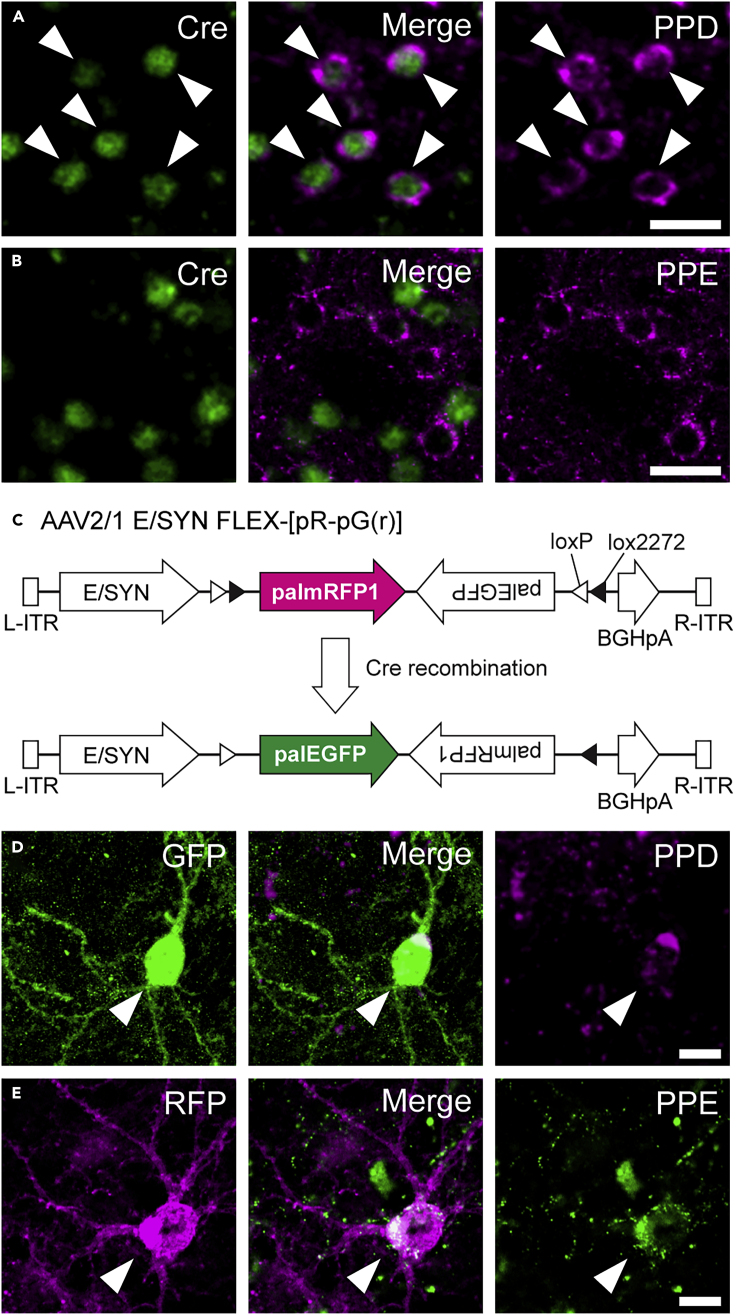
Specific Expression of GFP and RFP in Direct or Indirect Pathway Neurons (A and B) Double immunostaining for Cre recombinase and PPD or PPE in the CPu of Drd1-Cre transgenic mice. Immunoreactivity for Cre recombinase was observed in almost all PPD-immunoreactive cells (arrowheads) but not in PPE-positive cells. Scale bars: 20 μm. (C) AAV2/1-E/SYN-FLEX-[pR-pG(r)] vector. In the absence of Cre recombinase, the vector expresses palmitoylation site-attached mRFP1, palmRFP1 (pR). In contrast, the vector produces palmitoylation site-attached EGFP, palEGFP (pG), only in the presence of Cre recombinase by inverting the FLEX cassette. (D and E) Expression of reporter proteins in neostriatal neurons. One week after AAV vector injections, most GFP+ and RFP+ cells were immunoreactive for PPD and PPE, respectively. Arrowheads indicate double-positive neurons. Scale bars: 10 μm.

In order to label neostriatal neurons of the direct and indirect pathways with different fluorescent proteins, we applied the Cre-dependent recombination system with an AAV vector developed in this study and Cre-expressing transgenic mice. We first validated the specificity of Cre recombinase expression in dMSNs of Drd1-Cre transgenic mice using double immunofluorescence staining for Cre and chemical markers for dMSNs and iMSNs. MSNs are inhibitory GABAergic neurons and account for over 95% of neostriatal neurons (Graveland and DiFiglia, 1985; Rymar et al., 2004). dMSNs and iMSNs can be characterized based on their expression of chemical markers as well as their projection targets: dMSNs express Drd1 and preprodynorphin (PPD; the precursor protein of dynorphin), whereas iMSNs express dopamine receptor D2 (Drd2) and preproenkephalin (PPE) (Gerfen et al., 1990; Gerfen and Young, 1988; Le Moine and Bloch, 1995; Le Moine et al., 1990; Lee et al., 1997; Sonomura et al., 2007). We observed specific Cre recombinase expression in dMSNs in Drd1-Cre mice (Figures 1A and 1B). Almost all Cre-positive (+) neurons exhibited immunoreactivity for PPD (98.8%; 255 of 258 cells, n = 3 mice), and 95.5% of PPD+ neurons displayed immunoreactivity for Cre (255 of 267 cells) (Figure 1A). In contrast, only 3.6% of PPE+ neurons were immunoreactive for Cre (10 of 277 cells). Conversely, 3.6% of Cre+ neurons displayed immunoreactivity for PPE (10 of 280 cells) (Figure 1B).

We developed an AAV vector that expresses palmitoylation site-attached GFP (pG) or palmitoylation site-attached RFP (pR) in the presence or absence of Cre recombinase, respectively (Figure 1C). Focal injections of the AAV vector into the CPu of Drd1-Cre mice should thus label neighboring dMSNs and iMSNs with GFP and RFP, respectively. We tested the developed labeling system with immunofluorescence staining for GFP, RFP, PPD, and PPE in Drd1-Cre mice injected with the AAV vector. One week after injections, most GFP+ neurons were immunopositive for PPD (95.2%; 99 of 104 cells, n = 3 mice) and a few for PPE (2.9%; 3 of 101 cells). In contrast, most RFP+ neurons showed immunoreactivity for PPE (97.1%; 100 of 103 cells) but rarely for PPD (5.0%; 5 of 100 cells) (Figures 1D and 1E). In addition, both GFP+ and RFP+ neurons exhibited a high density of spines on their dendrites, a key feature of MSNs (Chang et al., 1981; Preston et al., 1980) (Figures 1D and 1E). These findings indicate that dMSNs and iMSNs were successfully labeled with GFP and RFP, respectively, following injections of the AAV vector into the CPu of Drd1-Cre transgenic mice.

### Visualization of dMSN and iMSN Axon Fibers by Immunoperoxidase Staining

**Figure 2:**
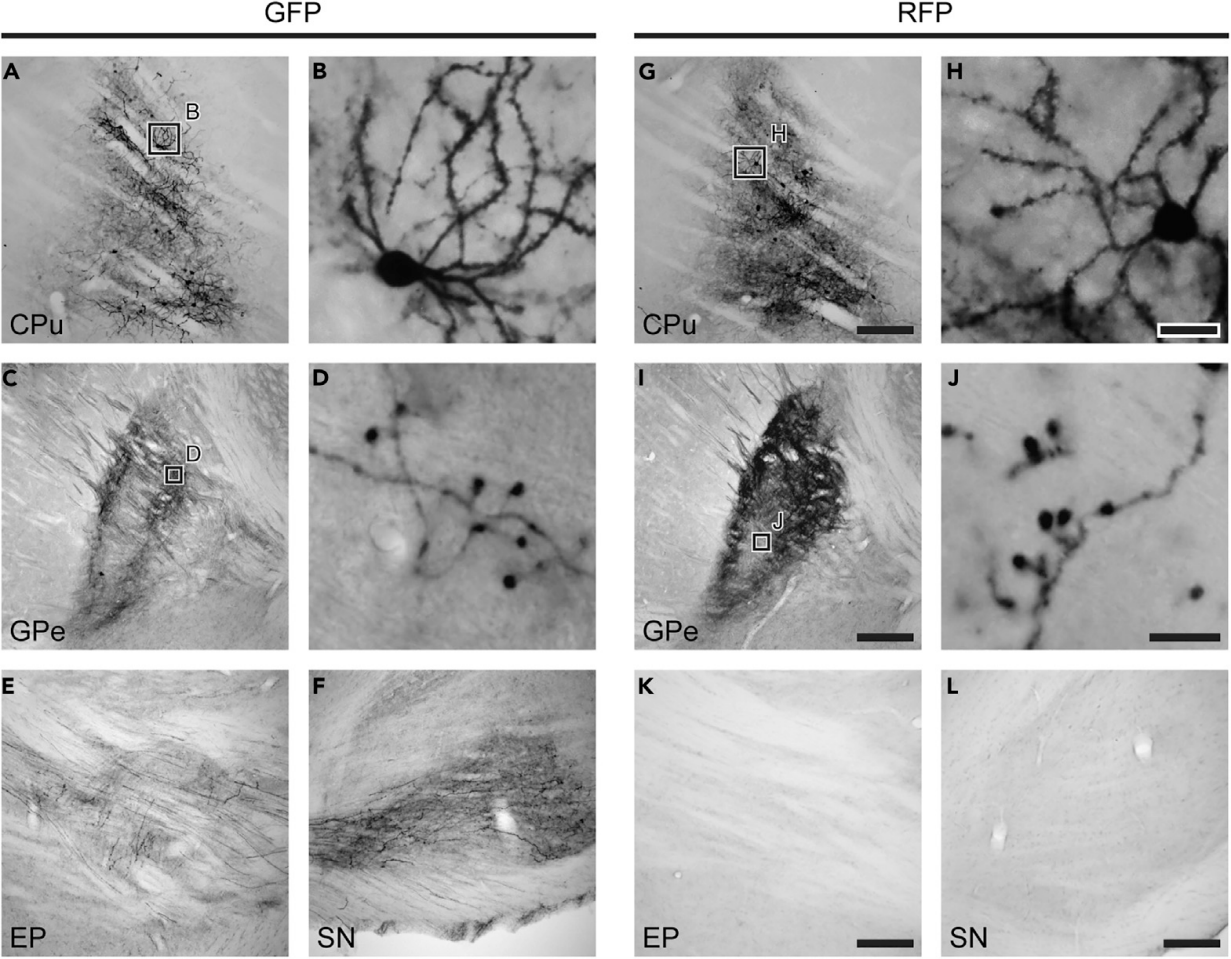
Labeling of Direct or Indirect Pathway Neurons by the AAV Vector (A and G) Injection sites of the AAV vector. The infected area was restricted to the CPu. Scale bars: 200 μm. (B and H) Higher magnification images of infected neurons in (A) and (G). Dendritic spines were clearly observed in both GFP+ and RFP+ neurons; these neurons were thus considered to be medium-sized spiny neurons (MSNs). Scale bars: 20 μm. (C, D, I, and J) GFP and RFP immunoreactivities in the GPe. Both GFP+ and RFP+ fibers were observed in the GPe. Boutons were clearly observed in GFP+ and RFP+ fibers. Scale bars: 200 μm (C and I) and 10 μm (D and J). (E, F, K, and L) GFP and RFP immunoreactivities in the EP and SN. GFP+ fibers were observed in the EP and SN, whereas RFP+ fibers were rarely detected in these output nuclei. Scale bars: 200 μm. See also Figure S1.

To visualize axon fibers with high sensitivity, we performed immunoperoxidase staining for GFP and RFP in mouse brain sections (n = 3 mice). In the CPu, strong immunoreactivities for GFP and RFP were observed in cell bodies, dendrites, and axons (Figures 2A and 2G). Furthermore, dense spines were clearly observed on their dendrites (Figures 2B and 2H). RFP+ aspiny neurons were occasionally observed (data not shown). These aspiny interneurons do not express Cre recombinase, and thus they were labeled with RFP when the present method was used. Given that axons of striatal interneurons remain within the CPu (Alexander and Crutcher, 1990), RFP+ axon fibers outside the CPu must originate from iMSNs in the CPu.

Consistent with a previous report (Albin et al., 1989), we observed GFP+ axon fibers in the EP and SNr (Figures 2E and 2F) and RFP+ fibers in the GPe (Figure 2I). Furthermore, we observed that axon collaterals of dMSNs were distributed in the GPe as well (Figures 2C and 2D). The GPe contained a substantial number of GFP+ axonal fibers (Figure 2C), which were not merely passing fibers of dMSNs, as axon varicosities were clearly observed in GFP+ axon fibers in the GPe, as well as in RFP+ fibers (Figures 2D and 2J). Recently, it has been reported that there is direct projection from the rat neocortex to the GPe (Karube et al., 2019). We confirmed that brain regions other than the CPu, such as the neocortex and thalamus, were not infected in the present study (Figure S1). A few RFP+ axon fibers were observed in the EP and SNr (Figures 2K and 2L). This is in accordance with a previous report, in which only 1.0% of PPE+ neurons projected to the SN (Lee et al., 1997).

### Distinct Axonal Arborizations Formed by dMSNs and iMSNs in the GPe

**Figure 3:**
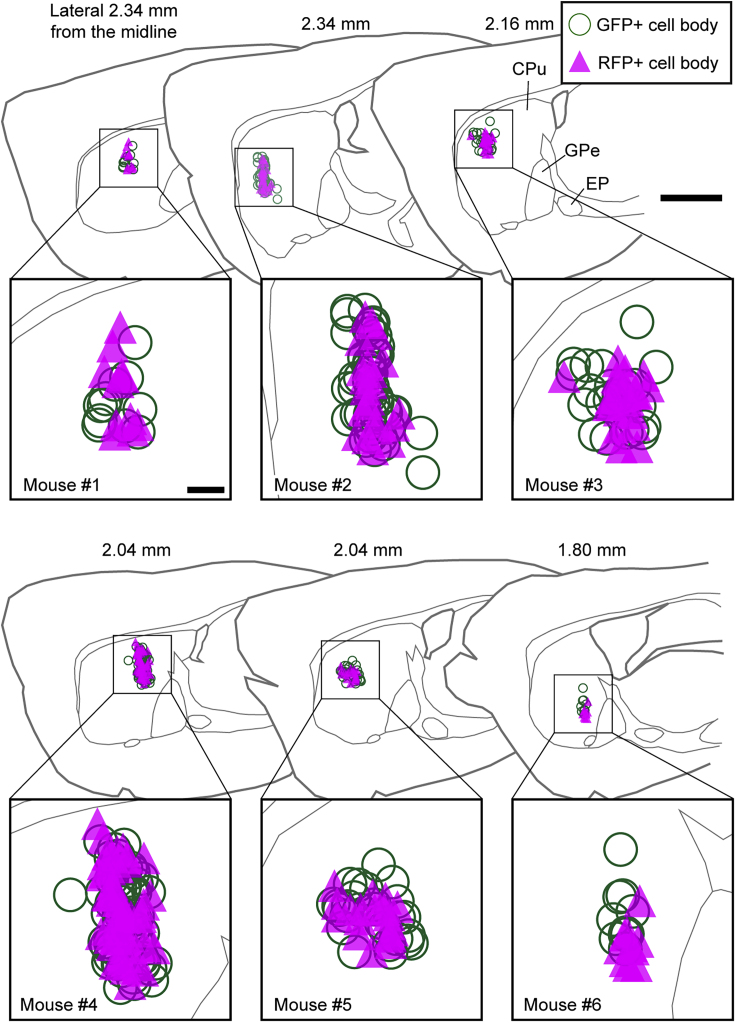
Distribution of GFP+ or RFP+ MSN Somata The locations of GFP+ or RFP+ cell bodies were superimposed onto parasagittal sections exhibiting the highest infection density in each mouse. Green circles and magenta triangles indicate GFP+ and RFP+ cell bodies, respectively. Scale bars: 1 mm and 200 μm (insets).

We investigated the distributions of GFP+ and RFP+ axon fibers in the GPe by AAV vector injections into the CPu of Drd1-Cre mice. We first quantified the number of GFP+ and RFP+ MSNs in each mouse and superimposed the locations of cell bodies in the parasagittal plane (Figure 3). The numbers of GFP+ and RFP+ MSNs were 28.7 ± 17.3 and 22.8 ± 14.0, respectively (mean ± SD, n = 6 mice). The cell bodies of GFP+ and RFP+ MSNs were distributed in the range of 0.52 ± 0.13 and 0.44 ± 0.11 mm in the rostrocaudal plane, 0.80 ± 0.15 and 0.72 ± 0.19 mm in the dorsoventral plane, and 0.53 ± 0.08 and 0.48 ± 0.10 mm in the mediolateral plane, respectively (mean ± SD, n = 6 mice). There was no obvious difference in the distributions of GFP+ and RFP+ cell bodies in the CPu.

**Figure 4:**
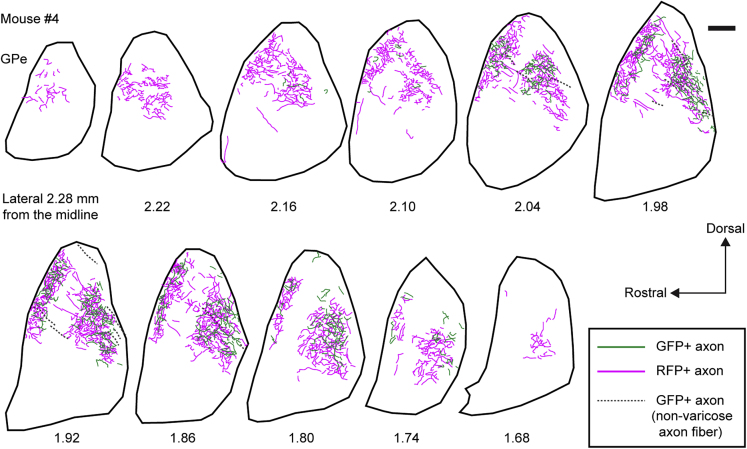
Projections of GFP+ or RFP+ Fibers to the GPe Green and magenta lines indicate GFP+ and RFP+ varicose fibers, respectively, in mouse #4. GFP+ fibers without varicosities are indicated by gray lines. All RFP+ fibers were varicose. Both GFP+ and RFP+ varicose fibers formed two axonal arborizations in the parasagittal plane. Widespread RFP+ fibers were observed in the GPe, whereas the distribution of GFP+ fibers was almost completely confined within that of RFP+ fibers. Scale bar: 200 μm. See also Figure S2.

After tracing GFP+ and RFP+ axon fibers in separate sections, two adjacent sections were superimposed in the parasagittal plane (Figures 4 and S2). RFP+ axon fibers were distributed over a wider range and at a higher density than that of GFP+ axon fibers. The projection fields of GFP+ and RFP+ axon fibers in the GPe were 0.63 ± 0.10 and 0.65 ± 0.13 mm in the rostrocaudal plane, 0.81 ± 0.12 and 0.92 ± 0.12 mm in the dorsoventral plane, and 0.62 ± 0.22 and 0.87 ± 0.16 mm in the mediolateral plane, respectively (mean ± SD, n = 6 mice). Thus, iMSNs exhibited wider and denser innervation of the GPe than that of dMSNs.

**Figure 5:**
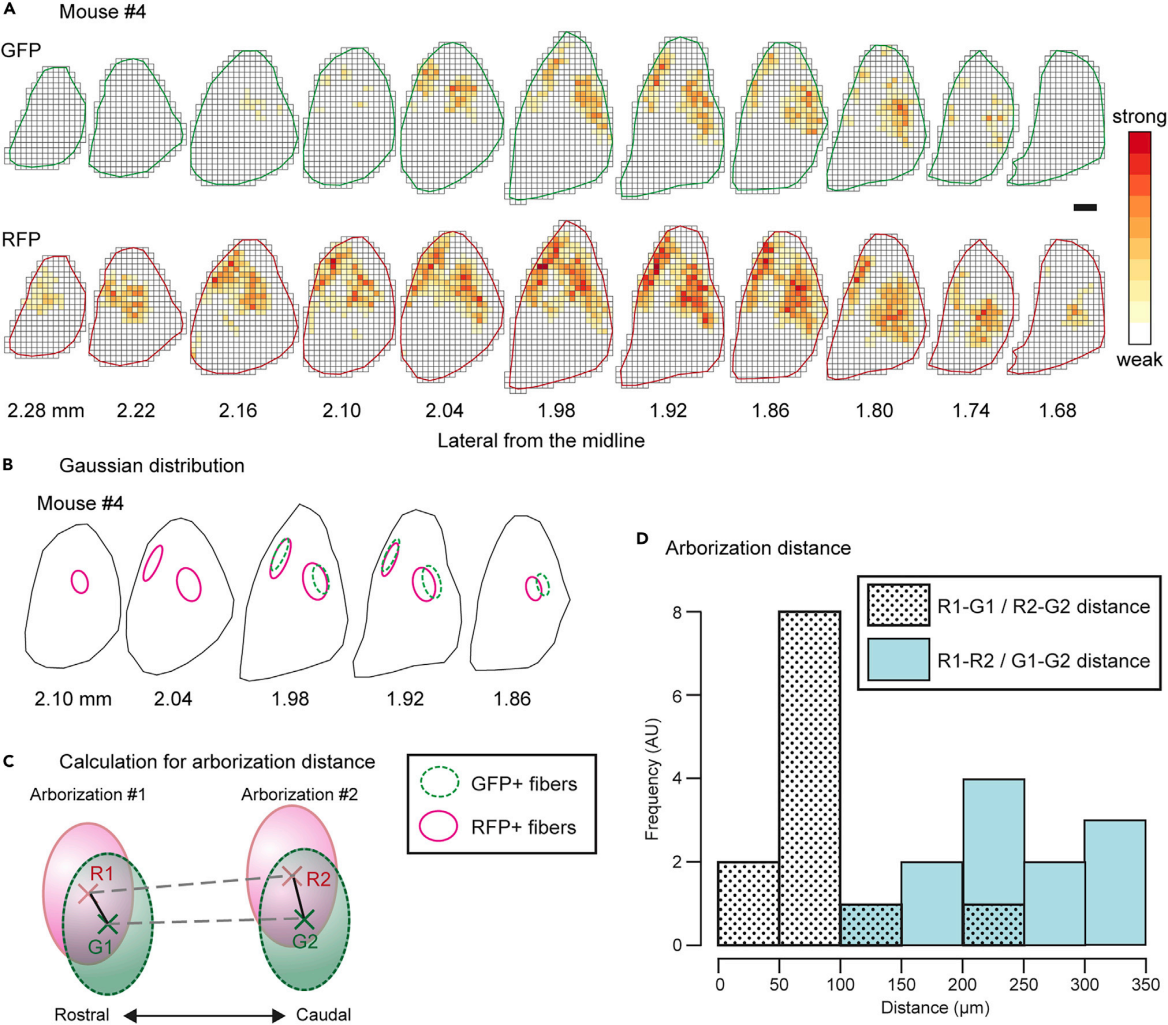
Density Analysis of GFP+ and/or RFP+ Axon Fibers in the GPe (A) Heatmap of fiber density in mouse #4. Pseudocolor represents the length of the varicose fibers in each box (40 μm × 40 μm). RFP+ fibers displayed a wider distribution and higher density than that of GFP+ fibers throughout the GPe. Scale bar: 200 μm. (B) Modeling of the density of axonal fibers by a mixture of two 3D Gaussian distributions in mouse #4. The green and magenta ellipses correspond to points with a Mahalanobis distance of 1 from the center of the Gaussian distributions fitted to GFP+ and RFP+ fibers, respectively. (C) Calculation of the Euclidean distances between the centers of the Gaussian distributions fitted to the arborizations. The crosses (G1, G2, R1, and G2) are the centers of the Gaussian distributions fitted to GFP+ (green) and RFP+ (magenta) fibers. Arborizations #1 (G1 and R1) and #2 (G2 and R2) represent the centers of the rostral and caudal arborizations. (D) Histogram of the distance between the axonal arborizations. R1-R2 and G1-G2 distances were significantly larger than R1-G1 and R2-G2 distances, indicating that both GFP+ and RFP+ fibers formed two axonal arborizations in the GPe, and the centers of GFP+ and RFP+ axonal arborizations mostly overlapped (p = 0.00002875, Kolmogorov-Smirnov test; n = 6 mice). See also Figures S3 and S4.

We subsequently analyzed the axonal distributions of dMSNs and iMSNs in the GPe. Both RFP+ and GFP+ fibers formed two axonal arborizations in the parasagittal plane, although the two arborizations were not clear in the trace image of mouse #3 (Figure S2). To determine the centers of the projection fibers, the densities of GFP+ and RFP+ varicose fibers in the GPe were quantified. We divided the GPe into 40 μm × 40 μm boxes in each parasagittal section and calculated the length of varicose axon fibers in each box, from which we generated a heatmap of axon density (Figures 5A, S3, and S4). The map reflected wider and denser distributions of RFP+ varicose fibers than GFP+ fibers, as shown in the trace images. The densities of GFP+ and RFP+ varicose fibers were individually fitted with two three-dimensional (3D) Gaussian distributions. We estimated regions where the Mahalanobis distance from the center was less than 1, which contains approximately 20% of the points drawn from the fitted Gaussian distribution. The distributions of GFP+ and RFP+ varicose fibers mostly overlapped (Figures 5B, S3, and S4). Next, the center of each Gaussian distribution was determined, and the rostral and caudal positions were named as Arborizations #1 and #2, respectively. These two 3D Gaussian fittings could likely represent two arborizations of MSNs in the GPe, even for mouse #3 (Figure S3). The distances between arborizations, which were defined as the Euclidean distances between the centers of the Gaussian distributions fitted to the arborizations, were calculated (Figure 5C). The distance between two GFP+ or RFP+ arborizations (G1-G2 or R1-R2) was significantly greater than the distance between overlapping GFP+ and RFP+ arborizations (G1-R1 or G2-R2) (Figure 5D; n = 6 mice). Collectively, these findings indicated that dMSNs and iMSNs formed two axonal arborizations in the GPe and that the center locations that dMSN and iMSN axon fibers targeted were almost overlapped in the GPe.

### Overlapping Projection Areas of dMSNs and iMSNs in the GPe

**Figure 6:**
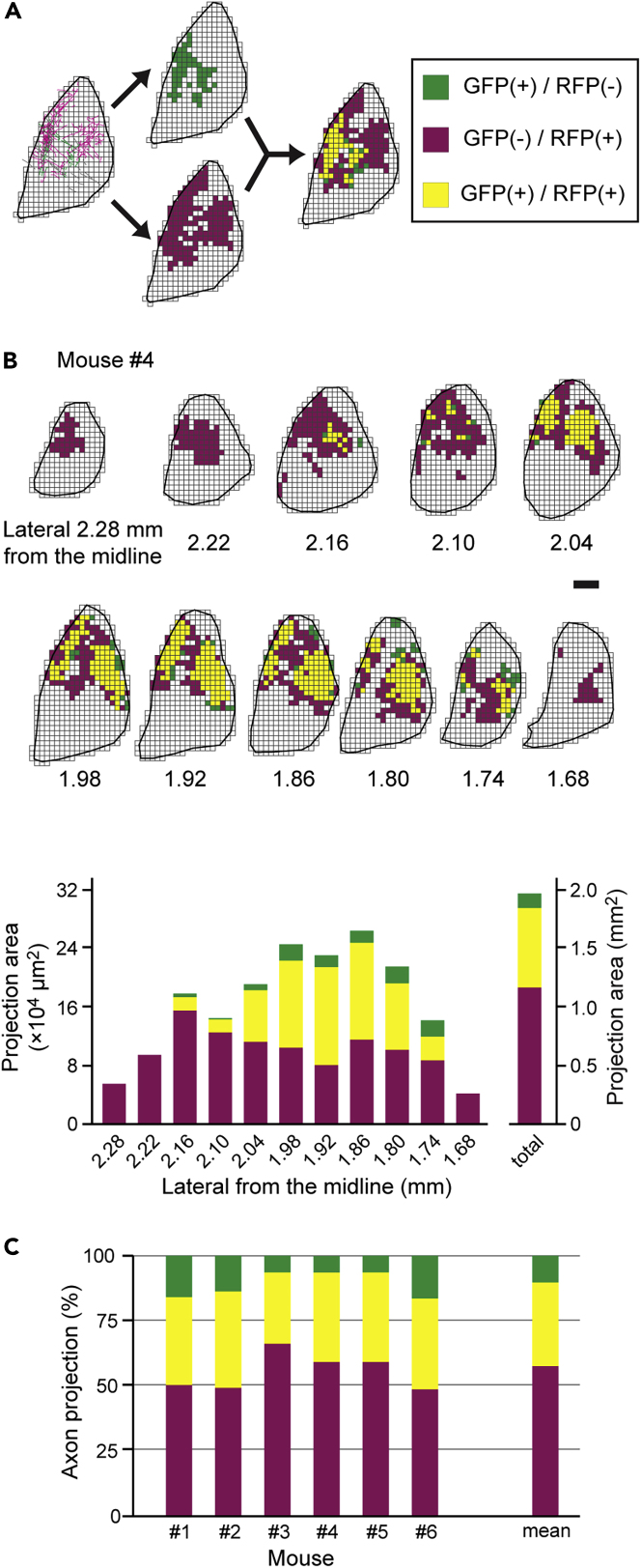
Area Analysis of GFP+ and/or RFP+ Axon Fiber Distribution in the GPe (A) Method of analysis of the axon projection area. The GPe was subdivided into 40 μm × 40 μm boxes, and axonal distribution was binarized depending on whether they contained GFP+ or RFP+ fibers, shown in green or red, respectively. The yellow boxes represent areas containing fibers that were positive for both GFP and RFP. Scale bar: 200 μm. (B) An example of the area analysis in mouse #4. The number of each box type was counted in parasagittal sections and converted to the area [×10^4^ μm^2^]. Each of these areas [×10^4^ μm^2^] and their sums [mm^2^] are represented by a bar graph. Note that the “total” here is the sum of the areas calculated for the analyzed sections and does not represent the total number of actual axon projection fields. (C) Each bar chart shows the percentage of the total number of each box type in six mice. The rightmost bar indicates the average of all mice. GFP+ fibers converged in RFP+ projection areas. Green, red, and yellow bars indicate boxes containing GFP+, RFP+, and both GFP+/RFP+ fibers. See also Figure S5.

In addition to the target centers of MSNs in the GPe, we analyzed the projection area of GFP+ and/or RFP+ axon fibers in the GPe, focusing on the area where the axons were distributed rather than on axon density. We divided the GPe into 40 μm × 40 μm boxes and classified individual boxes based on the presence of GFP+ and/or RFP+ varicose fibers (Figure 6A). In the lateral and medial sections of each mouse, RFP+ varicose fibers were predominant, whereas GFP+ fibers were rarely observed (Figures 6B and S5). Conversely, in the middle sections located between the lateral and medial sections of each mouse, the projection area of GFP+ fibers increased, mostly overlapping with the area containing RFP+ fibers. The number of boxes were counted in each parasagittal section, and the percentage of GFP+ and/or RFP+ projection areas in all sections was determined (Figure 6C). In all mice, half of the axon projection areas were positive only for RFP, showing that iMSNs projected to the GPe over a wider range than dMSNs. Most GFP+ fiber projection areas were included in the RFP+ projection areas. The ratios of boxes positive for RFP only, GFP only, and both were 58.2 ± 10.7%, 10.2 ± 5.7%, and 31.5 ± 5.9%, respectively (mean ± SD, n = 6 mice). Thus, in the GPe, dMSN axon collaterals converged proximally in the center of the field that receives axonal projections from neighboring iMSNs.

### Topographical Projections of dMSNs and iMSNs to the GPe

**Figure 7:**
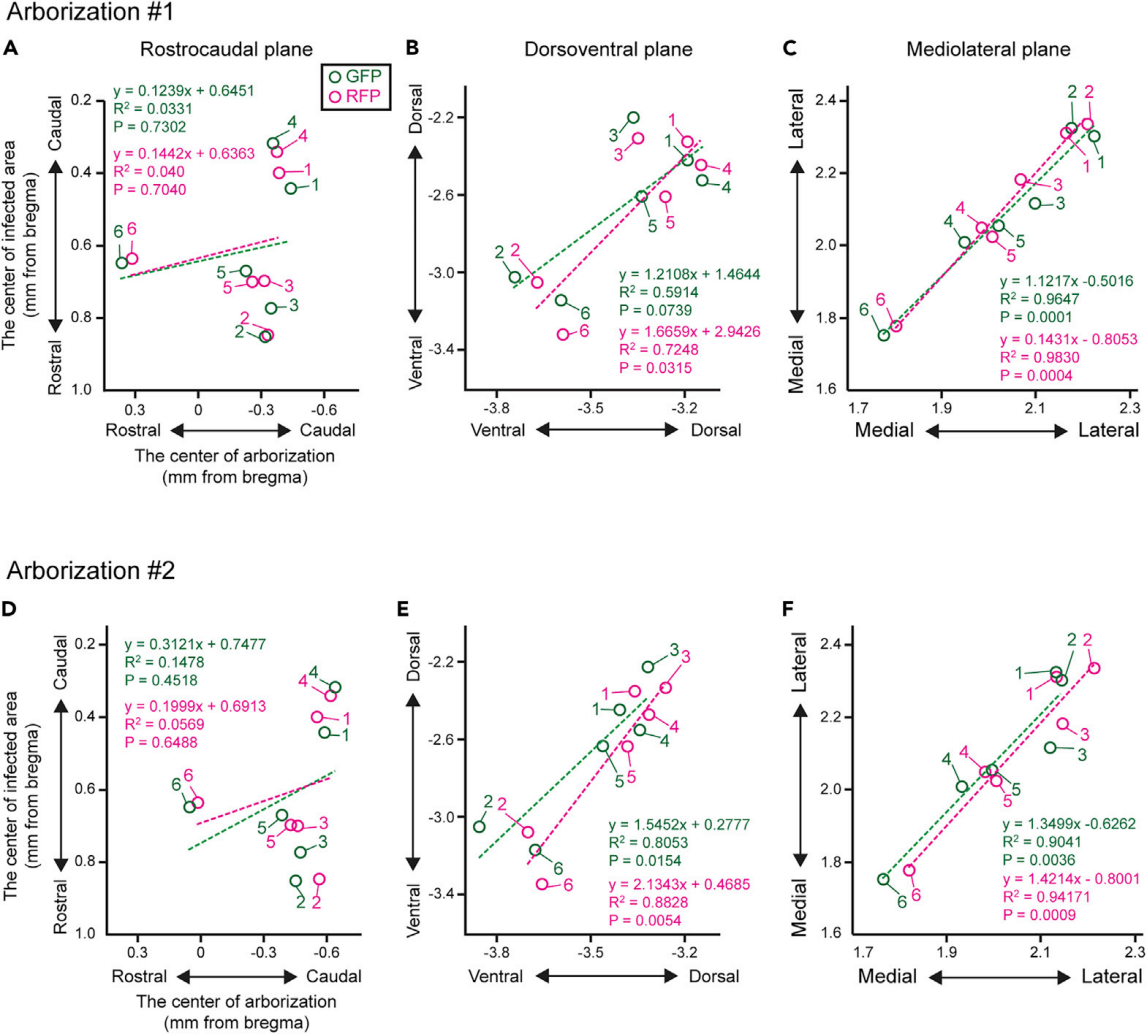
Topographical Projections of dMSNs and iMSNs to the GPe For Arborizations #1 and #2, the centers of the infected MSN somata and axonal arborizations are plotted along the rostrocaudal (A and D), dorsoventral (B and E), and mediolateral planes (C and F) (n = 6 mice). The vertical and horizontal axes represent the centers of the infected areas in the CPu and the axonal arborizations in the GPe, respectively. Green and magenta circles indicate the positions of GFP and RFP in each mouse. Dotted lines indicate linear regression lines. Strong correlations were found in the dorsoventral and mediolateral planes.

The fact that neighboring dMSNs and iMSNs sent axons to the nearby subfield in the GPe suggests topographical organization of striatopallidal pathway. We then investigated the relationship between the positions of the infected MSNs in the CPu and the distributions of axon fibers in the GPe. The centers of the infected soma distributions were calculated from Figure 3, and the centers of the axon projections were determined using Arborizations #1 and #2 estimated in Figure 5C (n = 6 mice). The 3D coordinates from bregma were determined with reference to the brain atlas (Franklin and Paxinos, 2007), and the centers of axonal arborizations were compared with those of soma distributions (Figure 7). In the rostrocaudal plane, no clear correlation was found in the present study (Figures 7A and 7D). In the dorsoventral plane, there was a strong correlation between the infection and projection sites in Arborization #2 (Figure 7E). In Arborization #1, the dorsoventral distribution of GFP+ axon fibers also tended to correlate with that of GFP+ cell bodies, even though this correlation was not statistically significant (Figure 7B). In the mediolateral plane, very strong correlations were detected in both Arborizations #1 and #2 (Figures 7C and 7F). This result demonstrates that the mediolateral and dorsoventral topography of neostriatal cell bodies are credible in both dMSN and iMSN axons targeting the GPe.

**Figure 8:**
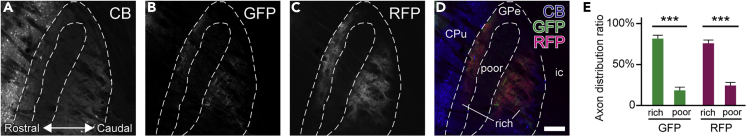
Overlap Distribution of CB Immunoreactivity and MSN Axon Fibers in the GPe (A–D) Distributions of CB, GFP, and RFP immunoreactivities in the GPe. One week after AAV vector injections into the CPu of Drd1-Cre mice, brain sections were immunostained for CB, GFP, and RFP. The GPe can be divided into two regions—CB-rich and CB-poor—according to the immunoreactivity for CB. Scale bar: 100 μm. (E) The total immunofluorescence intensities of GFP and RFP in CB-rich and CB-poor regions were normalized to 1 arbitrary unit in each mouse, and the respective percentages were calculated. Data are represented as mean ± SD. A significant difference was detected between the two regions in both GFP and RFP (Tukey's post hoc test; ∗∗∗p < 0.001; n = 3 mice).

Moreover, the striatopallidal circuit topography is known to be based on calbindin (CB) immunoreactivity, where MSNs in the CB-rich region in the CPu project to the CB-rich region in the GPe, and vice versa (Kita and Kita, 2001); the GPe has also been reported to be divided into two regions depending on CB immunoreactivity (Kita and Kita, 2001), which appeared to be similar to the distribution of MSN axonal arborizations revealed in the present study. Therefore, we finally examined the overlap between CB immunoreactivity and GFP+ and RFP+ axon fibers in the GPe. One week after AAV vector injections, we performed triple immunofluorescence staining for CB, GFP, and RFP. CB immunoreactivity was strong in the rostral region near the boundary between the CPu and GPe and in the caudal part of the GPe (CB-rich region), whereas the immunoreactivity was weak in the intermediate zone (CB-poor region) (Figure 8A). The immunoreactivities for GFP and RFP were strong in the CB-rich regions and weak in the CB-poor regions, consistent with changes in the intensity of CB immunoreactivity (Figures 8A–8D). When the fluorescence intensities of GFP+ and RFP+ axons were quantified, the axons of both cell types were significantly more abundant in the CB-rich region than in the CB-poor region (p < 0.001 for GFP+, p < 0.001 for RFP; n = 3 mice) (Figure 8E). Similar to the results of immunoperoxidase staining, fluorescence imaging revealed that dMSNs and iMSNs projected to the same regions of the GPe and that iMSNs had a wider projection range (Figures 8B–8D).

## Discussion

**Figure 9:**
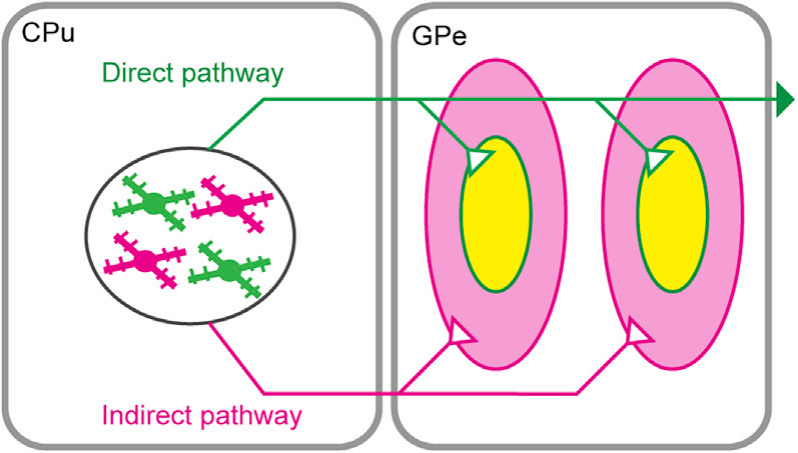
Schematic Diagram of Direct and Indirect Pathways in the GPe dMSNs and iMSNs are shown in green and magenta, respectively. Regions containing fibers positive for both GFP and RFP are displayed in yellow.

In this study, we characterized the axonal projection patterns of dMSNs as well as iMSNs in the GPe by using the AAV vector that selectively labels neighboring dMSNs and iMSNs with different fluorescent proteins. Both dMSNs and iMSNs formed two axonal arborizations in the rostral and caudal parts of the GPe and showed topographical projections in the dorsoventral and mediolateral planes. The axon fibers of dMSNs displayed a spatially limited distribution within the iMSN-targeted subfield in the GPe (Figure 9). This overlapping topography of the respective projections would contribute to linking information from direct and indirect pathway neurons within the GPe.

Although we analyzed the projections of dMSNs and iMSNs to the GPe using anterograde labeling with the AAV vector, we did not confirm the projections by retrograde tracing techniques in the present study. As iMSNs project to a wider range of the GPe and only a portion of dMSNs send axon collaterals to the GPe (Fujiyama et al., 2011), more iMSNs than dMSNs would be labeled by retrograde labeling. Indeed, it has been reported that injection of rhodamine dextran amine 3 kDa into the GPe labeled neostriatal neurons positive for Drd2 about four times more than neurons immunoreactive for Drd1 (Deng et al., 2006).

In Drd1-Cre transgenic mice, Cre recombinase is specifically expressed in dMSNs, enabling labeling of dMSNs with GFP by the flip-excision (FLEX) (Schnutgen et al., 2003) switch-containing AAV vector in the present study. As RFP is expressed in the absence of Cre recombinase, both iMSNs and interneurons were labeled with RFP. MSNs account for 90%–98% of all neurons in the CPu (Kawaguchi et al., 1995; Kemp and Powell, 1971; Rymar et al., 2004). The remaining neurons are cholinergic or GABAergic interneurons (Kreitzer, 2009). Since the cell bodies of cholinergic interneurons are large (Kawaguchi, 1993), they are easily distinguished from MSNs and other GABAergic interneurons by large differences in cell body size. Although the somata of GABAergic interneurons are similar in size to those of MSNs, they are characterized by non-spiny dendrites (Bennett and Bolam, 1993; Kawaguchi, 1993) and can be discriminated from MSNs by the absence of spines. When analyzing the number and distribution of the infected MSNs in the CPu, non-MSN cells were excluded based on these morphological features. In addition, since the axons of interneurons remain within the CPu, they did not confound the present analysis, which assessed the density and area of dMSN and iMSN axon fibers within the GPe. Therefore, we successfully evaluated the axon projections of dMSNs and iMSNs to the GPe.

Topographical projections of dMSNs and iMSNs to the GPe were clearly observed in the dorsoventral and mediolateral planes, a finding that was in good accordance with that of a previous study demonstrating similar topographical projections of a mixture of dMSNs and iMSNs (Gerfen, 1985). Using the method developed in the present study, we successfully labeled dMSNs and iMSNs separately and demonstrated that each of them projects topographically. The topographic organization of striatopallidal projections in the rostrocaudal plane has also been reported using an autoradiographic tracer (Wilson and Phelan, 1982). However, no clear correlation was detected between the infected MSN somatic positions and the axon fiber distributions in the present study. Regardless of the locations of the infected MSN somata in the CPu, the axon projections appeared to converge at approximately the same position in the GPe: Arborizations #1 and #2 were distributed in the range of −0.3 to −0.5 mm and −0.4 to −0.6 mm, respectively, except in mouse #6. It is possible that mouse #6, whose infected area was located most ventromedially, exhibited a different projection pattern. Further studies are required for comprehensive clarification of striatopallidal topography, especially in the rostrocaudal plane, using 3D quantitative analysis in large samples.

The GPe is known to be divided into two regions—CB-rich and CB-poor regions (Kita and Kita, 2001). We have demonstrated that both dMSNs and iMSNs send axons to the CB-rich region in the GPe. Furthermore, it has been reported that the CPu also contains CB-rich and CB-poor regions and that each makes reciprocal connections with the corresponding regions of the GPe (Kita and Kita, 2001). In the present study, no projection of MSNs to the CB-poor region in the GPe were noted, probably because CB-poor regions in the CPu are confined to a narrow dorsolateral region and the AAV vector did not infect the CB-poor regions in the CPu. It would be interesting to apply more sophisticated methods to comprehensively analyze the reciprocal connections between the corresponding regions in the CPu and GPe.

The present study demonstrated that dMSNs and iMSNs formed two axonal arborizations in the same regions of the GPe. These overlapping projections may form the structural basis for cooperative function of dMSNs and iMSNs, consistent with the emerging model of the basal ganglia (Peak et al., 2019). It has been proposed that axon collaterals of dMSNs in the GPe link information from direct and indirect pathway neurons within the GPe (Cazorla et al., 2014). Indeed, the excitability of iMSNs bidirectionally regulates the amount of dMSN axon collaterals in the GPe: chronic enhancement or suppression of iMSN excitability increased or decreased the amount of dMSN projections to the GPe, respectively, suggesting that plasticity may regulate the output balance from the neostriatum (Cazorla et al., 2014). It will be necessary to examine the mechanisms of axonal plasticity of dMSNs and iMSNs in the GPe in more detail, considering the convergent projections revealed in the present study.

Our present findings of convergence of dMSN axons in the central area of iMSN projections in the GPe extend the classical model of the basal ganglia. In the classical model, dMSNs enhance the activity of thalamic neurons through output nuclei (EP and SNr) and facilitate desired actions, whereas iMSNs suppress competing movements by inhibiting the thalamus (Albin et al., 1989; Mink, 1996). The axons of iMSNs, which spread more widely in the GPe, achieve surround suppression of thalamic neuron activity. Conversely, dMSNs may enhance the activity of thalamic neurons and strongly suppress thalamic activity with a slight time delay by axon collaterals to the GPe accelerating the indirect pathway. This feedforward inhibition may enhance temporal resolution of thalamic neuron activity.

### Limitations of the Study

In this study, the distribution of axon fibers in the GPe was analyzed using bright-field staining for GFP or RFP in 20-μm-thick adjacent sections, but all fibers were not visualized and quantified. In order to accurately estimate the length and distributions of GFP+ and RFP+ fibers in the GPe, the fibers should be fully reconstructed in 3D. Owing to the high density of the projecting fibers to the GPe, it is laborious to perform a full 3D reconstruction with thin sections, and it would be more efficient to observe the fibers three-dimensionally with thicker specimens, such as slices and hemispheres, using a tissue-clearing method.

Furthermore, we analyzed the projections of dMSNs and iMSNs using only Drd1-Cre mice but not Drd2- or A2a-Cre mice, which express Cre recombinase specifically in iMSNs in the CPu. The use of these strains would have led to more convincing conclusions about the overlapping projections of dMSNs and iMSNs to the GPe.

Finally, and most importantly, the current technique does not allow us to determine whether dMSNs and iMSNs target the same or different neurons in the GPe. Therefore, in order to elucidate the functional significance of the overlapping projections revealed in this study, it is important to identify the types of GPe neurons that receive synaptic inputs from dMSNs and/or iMSNs in future studies.

### Resource Availability

#### Lead Contact

Further information and requests for resources and reagents should be directed to and will be fulfilled by the Lead Contact, Hiroyuki Hioki (h-hioki@juntendo.ac.jp).

#### Materials Availability

All unique biological materials generated in this study are available from the lead contact with a completed materials transfer agreement.

#### Data and Code Availability

All data and custom scripts used in this study are available upon request.

## Methods

All methods can be found in the accompanying Transparent Methods supplemental file.

## Supplemental Information

Document S1. Transparent Methods, Figures S1–S5, and Table S1
