# Supplementary material for: Overlapping Projections of Neighboring Direct and Indirect Pathway Neostriatal Neurons to Globus Pallidus External Segment
Source: iScience. 2020 Sep 1;23(9):101409. doi: 10.1016/j.isci.2020.101409 (PMC7520896; doi:10.1016/j.isci.2020.101409)
Supplement: Document S1. Transparent Methods, Figures S1–S5, and Table S1 [file mmc1.pdf]

iScience, Volume *23*

Supplemental Information

Overlapping Projections of Neighboring Direct and Indirect Pathway Neostriatal Neurons to Globus Pallidus External Segment

Shinichiro Okamoto, Jaerin Sohn, Takuma Tanaka, Megumu Takahashi, Yoko Ishida, Kenta Yamauchi, Masato Koike, Fumino Fujiyama, and Hiroyuki Hioki

Table S1. Primers used in the present study, Related to Figure 1.

P1	5′-CTAGCAAAATAGGCTGTCCCGAATTC**GCCACC**ATGCTGTG CTGTAT-3′
P2	5′-GATCCACTAGTTCTAGAGCGTTAGGCGCCGGTGGAGTGGC-3′
P3	5′-CGCTCTAGAACTAGTGGATCTTACTTGTACAGCTCGTCCA-3′
P4	5′-GACGATAGTCATGCCCCGCGGTCGAC**GCCACC**ATGCTGTGCTGTAT-3′
P5	5′-AAAACTCGAGTAGTTATTAATAGTAATCAA-3′
P6	5′-TTTTGGATCCCGCCGCAGCGCAGATGGTCG-3′

Bold characters indicate the Kozak consensus sequence. Underlined sequences indicate the restriction sites for *EcoR*I in P1, *Sal*I in P4, *Xho*I in P5, and *BamH*I in P6.

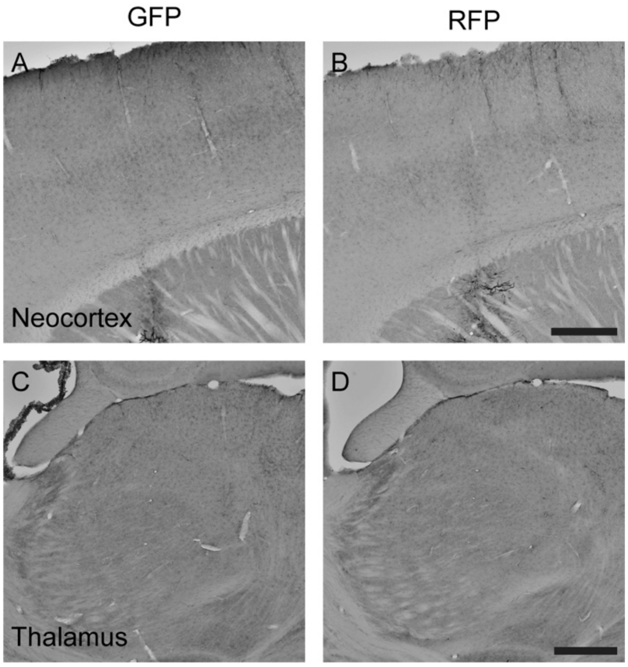

Figure S1. No infection of the AAV vector in the neocortex and thalamus, Related to Figure 2. (**A**, **B**) GFP and RFP immunoreactivities in the neocortex just above the injection site. The glass capillary passed through the neocortex during stereotaxic injection into the CPu, but there was no AAV infection in the neocortex (n = 6 mice). (**C**, **D**) GFP and RFP immunoreactivities in the thalamus. No immunoreactivity for GFP and RFP was observed in the thalamus (n = 6 mice). It was confirmed that there was no anterograde and/or retrograde infection in brain regions other than the CPu. Scale bars: 0.5 mm.

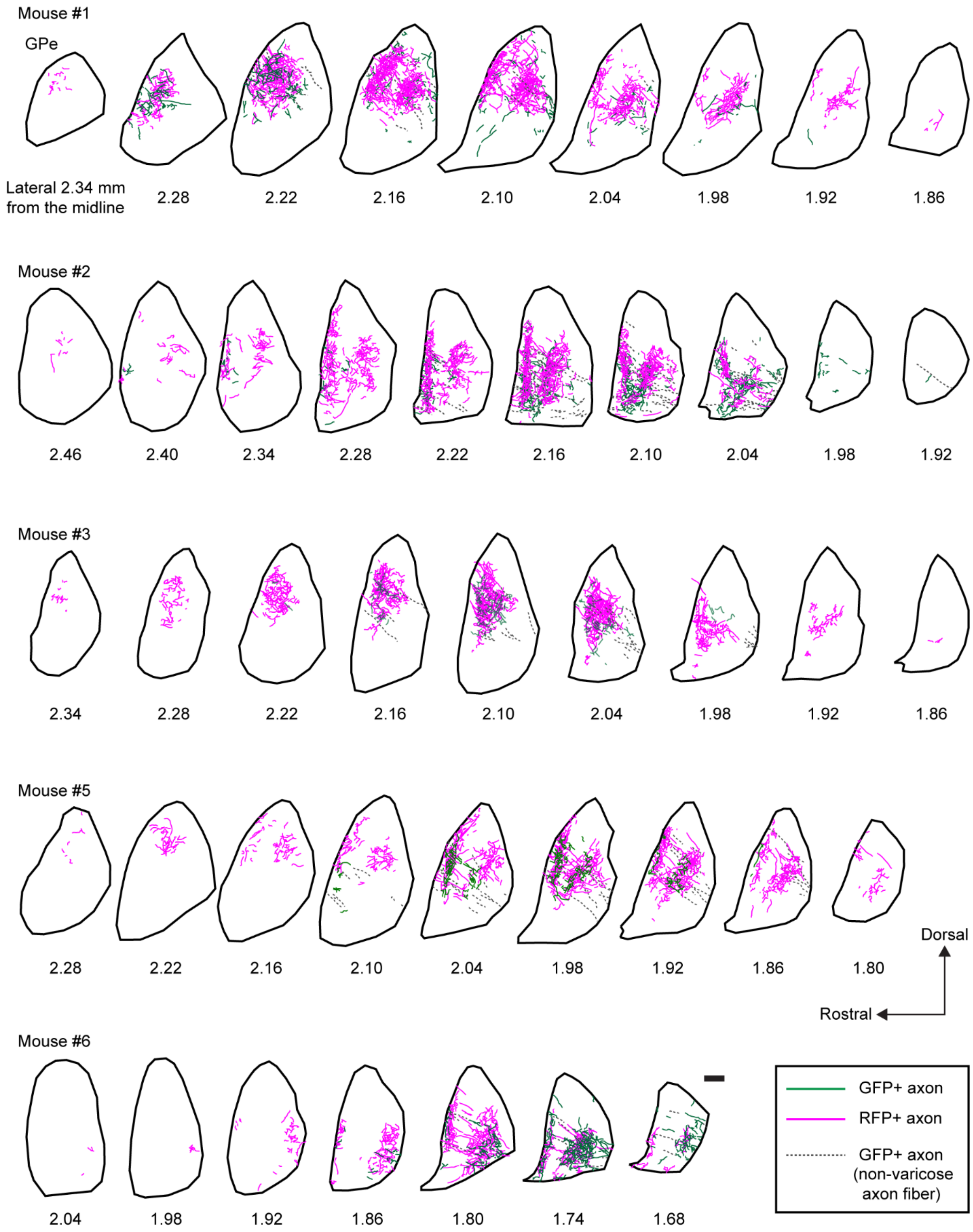

Figure S2. Projections of GFP+ or RFP+ fibers to the GPe, Related to Figure 4. Green and magenta lines indicate GFP+ and RFP+ varicose fibers, respectively, in mouse #1, #2, #3, #5, and #6. Fibers without varicosity were also found in GFP+ fibers (gray lines). Scale bar: 0.2 mm.

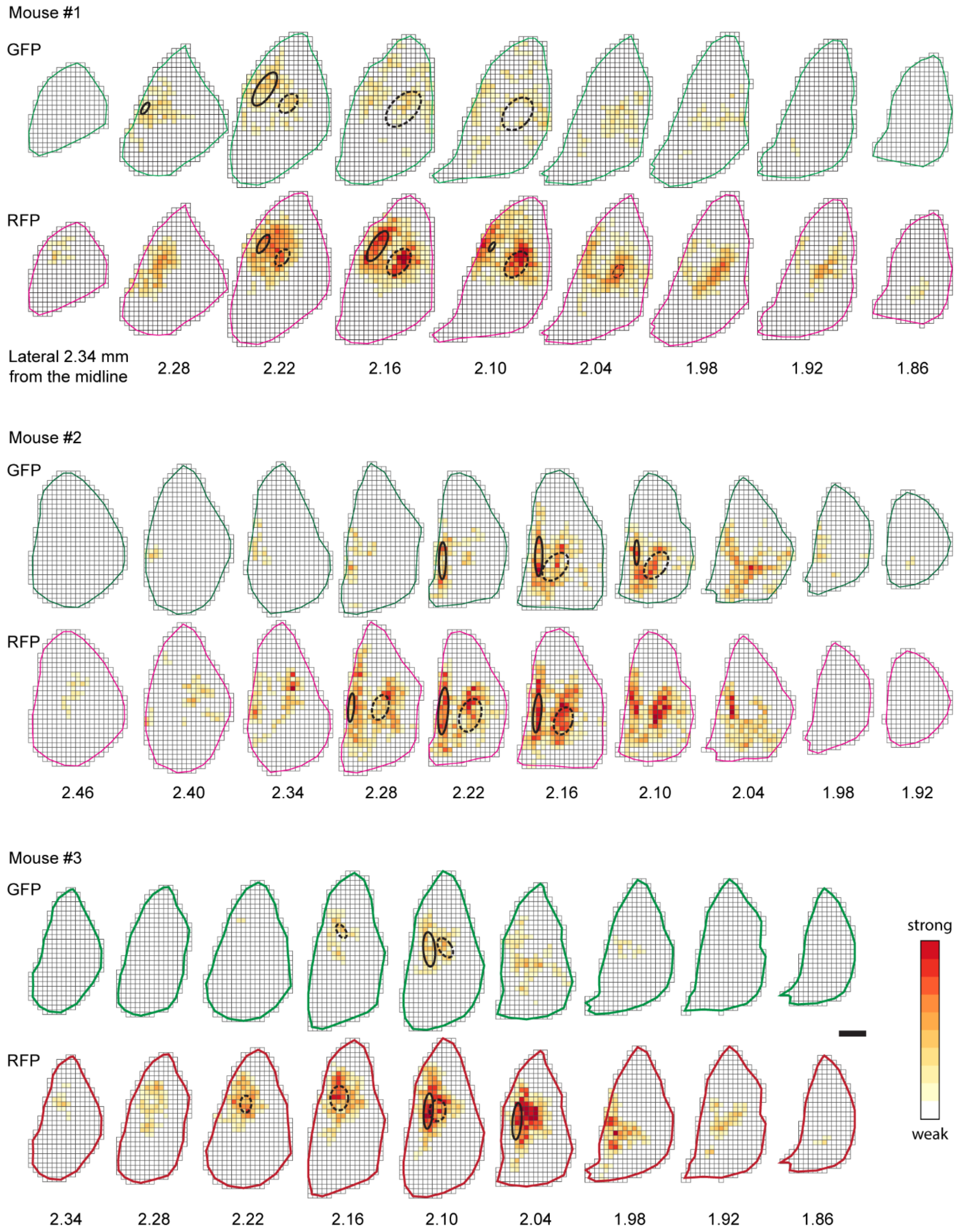

Figure S3. Density analysis of GFP+ and/or RFP+ axon fibers in the GPe in mouse #1, #2, and #3, Related to Figure 5. Fiber density heatmaps, where the pseudocolor represents the length of the varicose fibers in each boxed area (40 µm × 40 µm). Solid and dotted circles indicate Arborizations #1 and #2 located rostrally and caudally, respectively. Scale bar: 0.2 mm.

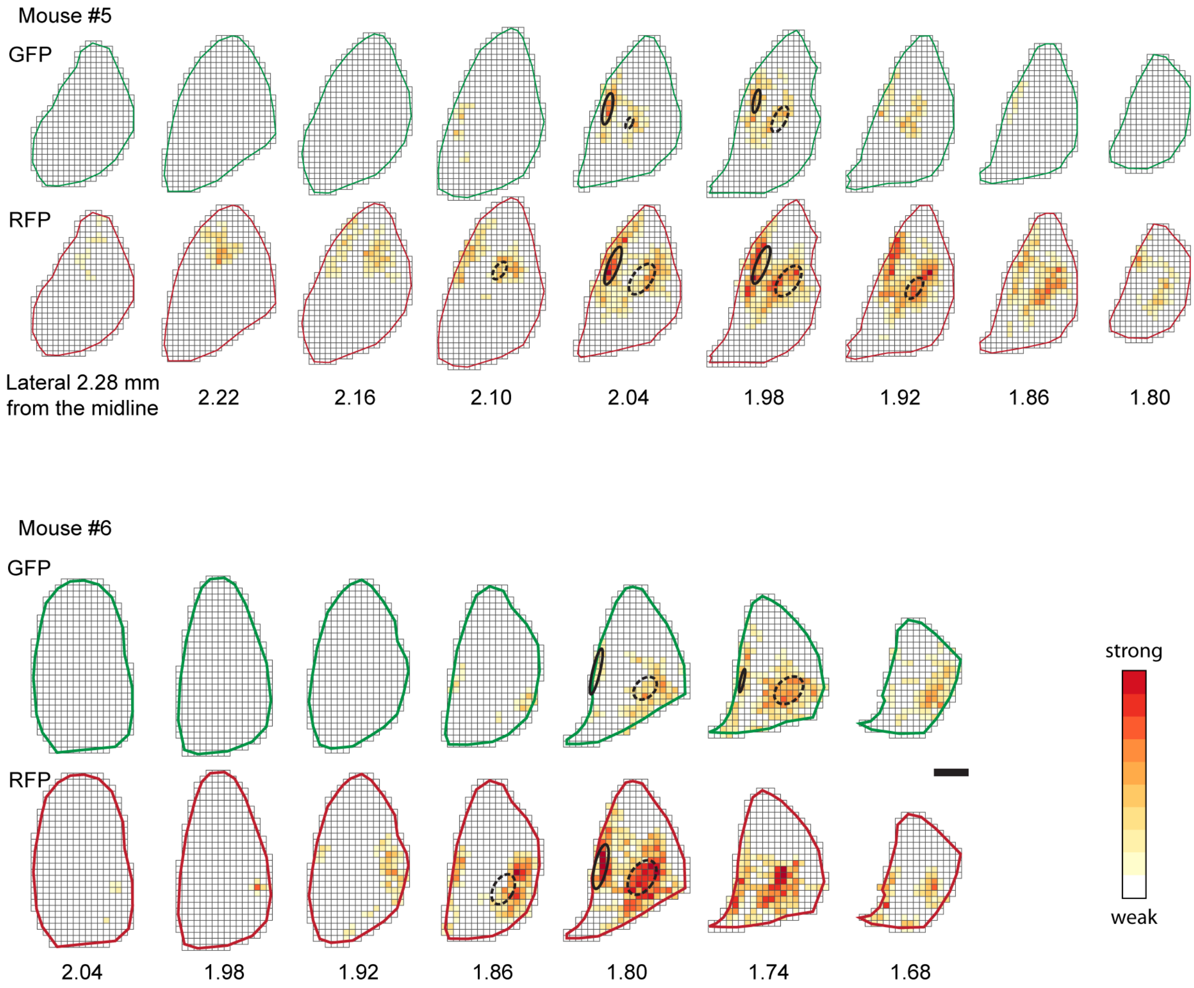

Figure S4. Density analysis of GFP+ and/or RFP+ axon fibers in the GPe in mouse #5 and #6, Related to Figure 5. Fiber density heatmaps, where the pseudocolor represents the length of the varicose fibers in each boxed area (40 µm × 40 µm). Solid and dotted circles indicate Arborizations #1 and #2 located rostrally and caudally, respectively. Scale bar: 0.2 mm.

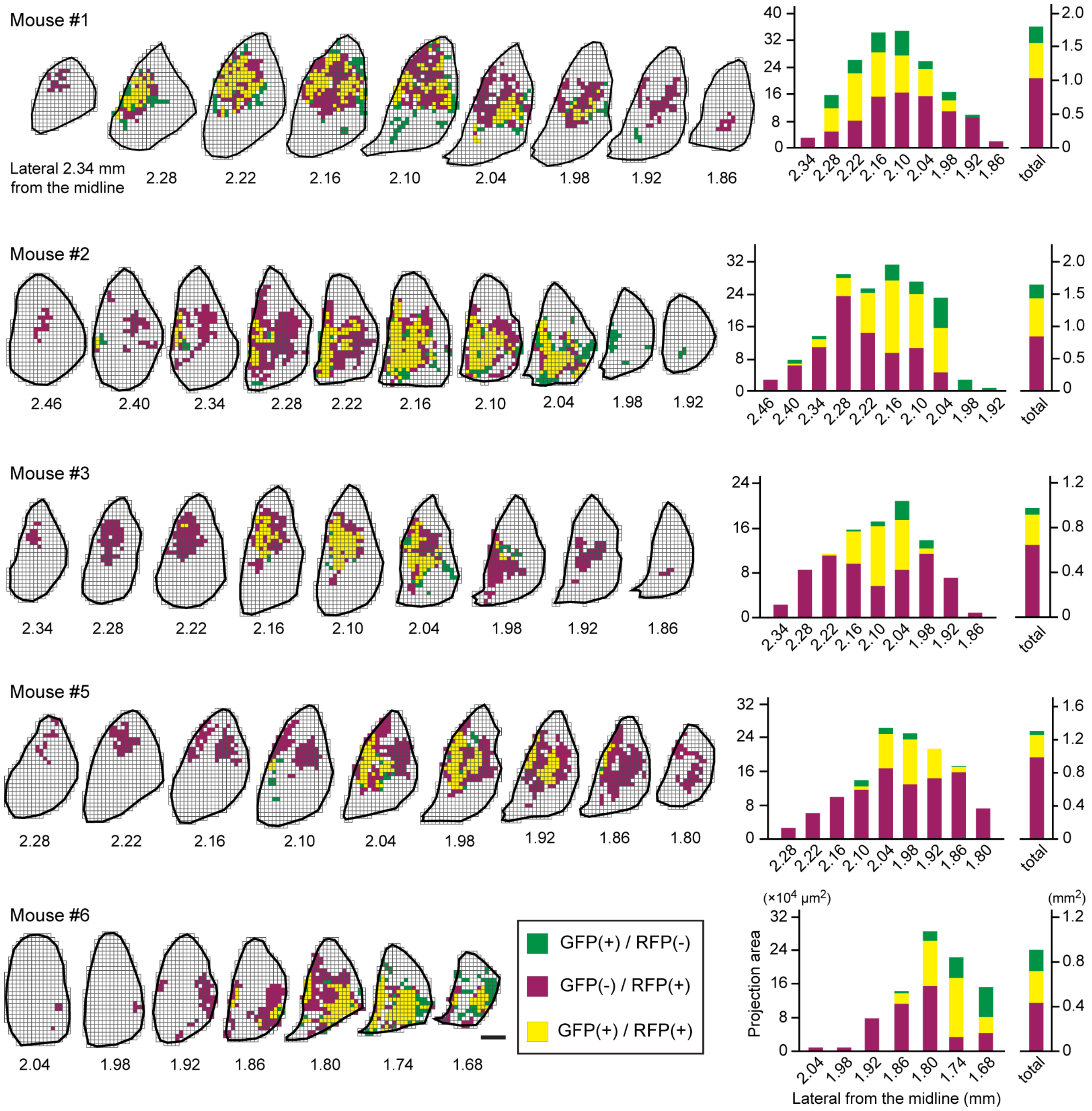

Figure S5. Area analysis of the distribution of GFP+ and/or RFP+ axon fibers in the GPe, Related to Figure 6. Green, red, and yellow boxes indicate the area containing the fibers that were positive for GFP only, RFP only, and both GFP and RFP, respectively. The number of each box type was counted in parasagittal sections and converted to the area [$\times 10^4$ μm^2]. Each of these areas [$\times 10^4$ μm^2] and their sums [mm^2] are represented by a bar graph. Note that the 'total' here is the sum of the areas calculated for the analyzed sections and does not represent the total number of actual axon projection ranges.

Transparent Methods

Animals

All procedures involving animals were in accordance with the National Institutes of Health Guide for the Care and Use of Laboratory Animals. The experiments were approved by the Committees for Animal Care and Use and those for Recombinant DNA Study at Kyoto University and Juntendo University. Drd1-Cre BAC transgenic male mice (8–16 weeks; n = 15 in total; FK150Gsat/Mmucd; stock number: 029178-UCD; MMRRC, UC Davis) (Gong et al., 2007) were used in the present study. Mice were maintained under a 12-h light/dark cycle and were provided *ad libitum* access to food and water. All efforts were made to minimize animal suffering and the number of animals used.

Fixation and tissue preparation

Drd1-Cre transgenic mice were deeply anesthetized by intraperitoneal injection of a three-drug mixture of medetomidine (0.3 mg/kg; Nihon Zenyaku Kogyo, Koriyama, Japan), midazolam (6 mg/kg; Sandoz, Tokyo, Japan), and butorphanol (Vetorphal®; 5 mg/kg; Meiji Seika Pharma, Tokyo, Japan). Mice were then perfused transcardially with 20 mL of 5 mM phosphate-buffered 0.9% saline (PBS; pH 7.4), followed by perfusion for 3 minutes with the same volume of 4% formaldehyde (16223-55; Nacalai Tesque, Kyoto, Japan), 75%-saturated picric acid (27925-25; Nacalai Tesque), and 0.1 M Na_2HPO_4 (adjusted to pH 7.2 with NaOH). The brains were removed and post-fixed overnight at 4ºC with the same fixative. After cryoprotection with 30% sucrose in 0.1 M sodium phosphate buffer (PB; pH 7.4), the brains were cut into 20-µm-thick sagittal sections on a freezing microtome (SM2000R; Leica Biosystems, Wetzlar, Germany). Sections were collected in 6 bottles containing 0.02% sodium azide in PBS and stored at 4°C until use for free-floating immunostaining. The shrinkage effect due to chemical fixation was not corrected for comparison of soma and axon distributions between dMSNs and iMSNs. When we measured the maximum lengths of the GPe along the rostrocaudal and dorsoventral planes, the GPe size changed by about 4% (rostrocaudal, 1.15 ± 0.03 mm; dorsoventral, 1.39 ± 0.04 mm; mean ± SD; n = 6 mice). For the analysis of topography in Figures 7 and 8, we normalized the fixed samples to the brain atlas (Franklin and Paxinos, 2007) to correct the shrinkage effect and determine the

coordinates with ADOBE ILLUSTRATOR CS3 software (Adobe Systems, San Jose, CA).

Production of AAV vector

We used the flip-excision (FLEX) switch (Schnutgen et al., 2003) to express GFP or RFP in the presence or absence of Cre. The sequence of a reporter protein that contained mRFP1 (Campbell et al., 2002) and inverted EGFP (Clontech, Palo Alto, CA), both tagged with a membrane-targeting signal, palmitoylation signal (Furuta et al., 2001; Kameda et al., 2008; Moriyoshi et al., 1996; Okada et al., 1999; Tamamaki et al., 2000) derived from GAP-43 N-terminus, was amplified by overlap PCR (primer set P1–P4; Table S1). The PCR product, pR-pG(r), was inserted into the *EcoRI*/*SalI* sites of pSK-hFLEX (Sohn et al., 2017), resulting in pSK-FLEX-[pR-pG(r)]. The *BamHI*-to-*MluI* fragment from the plasmid was complementarily inserted into the *MluI*/*BamHI* sites of an entry vector, p1A-TGB(r) (Hioki et al., 2009). Subsequently, we amplified the enhanced human synapsin I promoter (E/SYN) (Hioki et al., 2007) by PCR (primer set P5/P6; Table S1) and inserted the PCR product into the *XhoI*/*BamHI* sites of the plasmid, resulting in p1A-E/SYN-FLEX-[pR-pG(r)]. The entry vector was finally converted to pAAV2-E/SYN-FLEX-[pR-pG(r)] by homologous recombination with pAAV2-DEST(r) (Sohn et al., 2017) using LR clonase II (11791020; Thermo Fisher Scientific, Waltham, MA).

Production and purification of AAV vector particles were performed as reported previously (Hamamoto et al., 2017; Kataoka et al., 2014; Suzuki et al., 2015). Briefly, pAAV2-E/SYN-FLEX-[pR-pG(r)] and two helper plasmids, pBSIISK-R2C1 (Sohn et al., 2017) and pHelper (28060929; Stratagene, La Jolla, CA), were co-transfected into HEK293T cells using polyethylenimine (23966; Polysciences, Inc., Warrington, PA). Virus particles were extracted by three cycles of freeze-and-thawing, purified from the crude lysate of the cells by ultracentrifugation with OptiPrep (1114542; Axis-Shield, Oslo, Norway), and then concentrated by ultrafiltration with Amicon Ultra-15 (NMWL 50K; Merck Millipore, Darmstadt, Germany). The virus titer was adjusted to 1.0×10^{11} infectious units/mL (IFU/mL) with Dulbecco's phosphate-buffered saline (14249-95; Nacalai Tesque) containing 0.001 % Pluronic F-68 (24040032; Thermo Fisher Scientific). The virus solution was stored in aliquots at −80ºC until use for delivery to brain tissues.

Virus injection

Drd1-Cre transgenic male mice were deeply anesthetized by intraperitoneal injection of a mixture of medetomidine (0.3 mg/kg), midazolam (4 mg/kg), and butorphanol (5 mg/kg) and placed in a stereotaxic apparatus. We injected 0.2 μL of the virus solution into the CPu of Drd1-Cre mice by pressure through a glass micropipette attached to Picospritzer III (Parker Hannifin Corporation, Cleveland, OH). The injection coordinates were as follows: 0.5–1.0 mm anterior to bregma, 1.8–2.4 mm lateral to the midline, and 2.3–2.5 mm ventral to the brain surface. After the surgery, the mice received an intraperitoneal injection of atipamezole (Antisedan®; 1.5 mg/kg; Orion Corporation, Espoo, Finland) and recovered from anesthesia in about 15 minutes. The mice were maintained in specific pathogen-free conditions under a 12-hour light/dark cycle (light: 08:00–20:00) with *ad libitum* access to food and water for one week after AAV injections; subsequently, they were subjected to transcardial perfusion as described above.

Immunofluorescence labeling

After blocking with 10% normal donkey serum (NDS; S30-100ML; Merck Millipore) in PBS containing 0.3% Triton-X (35501-15; Nacalai Tesque) (PBS-X) for 30 minutes, the brain sections were incubated overnight with one of the following mixtures: (1) 1:5000 diluted rabbit anti-Cre recombinase antibody (69050; Merck Millipore) and 1 μg/mL of affinity-purified guinea-pig anti-PPD antibody (Lee et al., 1997); (2) 1:5000 diluted rabbit anti-Cre recombinase antibody and 1 μg/mL of affinity-purified guinea-pig anti-PPE antibody (Lee et al., 1997); (3) 20 μg/mL of chicken anti-GFP antibody (GFP-1020; Aves Labs, Tigard, OR) and 1 μg/mL of affinity-purified guinea-pig anti-PPD; (4) 1 μg/mL of affinity-purified rabbit anti-mRFP antibody (Hioki et al., 2010) and 1 μg/mL of affinity-purified guinea-pig anti PPE; or (5) 1:1000 diluted mouse anti-calbindin antibody (C9848; Merck Millipore), 20 μg/mL of chicken anti-GFP antibody, and 1 μg/mL of affinity-purified rabbit anti-mRFP antibody. The free-floating incubation was performed at 20–25ºC in PBS-X containing 0.12% λ-carrageenan (035-09693; Wako Chemicals, Osaka, Japan) and 1% NDS (PBS-XCD). Subsequently, the sections were incubated for 2 hours with one of the following mixtures: (a) 5 μg/mL of

AlexaFluor (AF) 488-conjugated antibody against rabbit IgG (A-11034; Thermo Fisher Scientific) and 5 μg/mL of AF568-conjugated antibody against guinea-pig IgG (A-11075; Thermo Fisher Scientific) for the primary antibody mixtures (1) and (2); (b) 5 μg/mL of AF488-conjugated antibody against chicken IgY (A-11039; Thermo Fisher Scientific) and 5 μg/mL of AF647-conjugated antibody against guinea-pig IgG (A-21450; Thermo Fisher Scientific) for the primary antibody mixture (3); (c) 5 μg/mL of AF568-conjugated antibody against rabbit IgG (A-11011; Thermo Fisher Scientific) and 5 μg/mL of AF647-conjugated antibody against guinea-pig IgG for the primary antibody mixture (4); or (d) 5 μg/mL of AF647-conjugated antibody against mouse IgG (A-21236; Thermo Fisher Scientific), 5 μg/mL of AF488-conjugated antibody against chicken IgY and 5 μg/mL of AF568-conjugated antibody against rabbit IgG for the primary antibody mixture (5). The sections were mounted onto gelatinized glass slides and coverslipped with 50% glycerol and 2.5% 1,4-diazabicyclo [2.2.2] octane (049-25712; Wako Chemical) in PBS.

The 3D image stacks were acquired under a TCS SP8 confocal laser scanning microscope (Leica Microsystems) equipped with a 25× water-immersion objective lens (HCX PL APO; NA = 0.95; Leica) and the pinhole set at 5.0 airy disk units. AF488, AF568, or AF647 were excited with 488, 543, or 643 nm laser beams and observed through 500–580, 590–650, or 660–850 nm emission prism windows, respectively. Images were then deconvolved with Huygens Essential software (version 3.8; Scientific Volume Imaging, Hilversum, Netherlands). Finally, the stack images were projected using ImageJ software (ver. 1.52i; National Institutes of Health).

Immunoperoxidase staining

All free-floating incubations were performed at 20–25ºC. After sectioning the brains with a freezing microtome, sections were serially collected in 6 bottles, as described above. The sections in the first and fourth bottles were incubated with 0.1 μg/mL of affinity-purified anti-GFP rabbit antibody (Nakamura et al., 2008; Tamamaki et al., 2000); those in the second and fifth bottles were incubated with 0.1 μg/mL of affinity-purified anti-mRFP rabbit antibody. The sections were incubated with a secondary antibody, biotinylated goat anti-rabbit IgG (BA-1000; Vector Laboratories, Burlingame, CA), in PBS-XCD for 2 hours, and then for 1 hour

with avidin-biotinylated peroxidase complex (1:100; ABC-elite; Vector Laboratories) in PBS-X. The sections were then incubated for 30 minutes in biotinylated tyramine (BT)-glucose oxidase (GO) reaction mixture containing 1.25 μM BT, 3 μg/mL of GO (16831-14; Nacalai Tesque), 2 mg/mL of beta-D-glucose (16804-32; Nacalai Tesque), and 1% bovine serum albumin (BSA; 01863-77; Nacalai Tesque) in 0.1 M PB (pH 7.4) (Furuta et al., 2009; Hioki et al., 2016; Kuramoto et al., 2009). The BT-GO method enables amplification of signals by the deposition of biotin molecules via the peroxidase activity of avidin-biotinylated peroxidase complex. Subsequently, the sections were again incubated for 1 hour with ABC in PBS-X. The bound peroxidase was finally developed by reaction for 30–60 minutes with 0.02% diaminobenzidine (DAB)-4HCl (347-00904; Dojindo, Kumamoto, Japan) and 0.0001% H_2O_2 in 50 mM Tris-HCl, pH 7.6. All stained sections were serially mounted onto gelatinized glass slides, dried, serially dehydrated in ethanol, cleared in xylene, and coverslipped with NEW MX mounting medium (FX00500; Matsunami, Kishiwada, Japan).

Axon tracing

DAB-stained sections were automatically captured as large color images using a TOCO digital slide scanner (CLARO, Aomori, Japan) equipped with a 10x objective lens (EC Plan-Neo fluar; NA, 0.30; Zeiss, Oberkochen, Germany). On the images, we traced and digitized the axonal fibers with a pen tablet (Bamboo Tablet; Wacom Corporation, Saitama, Japan) and ADOBE ILLUSTRATOR CS3 software.

Quantification and statistical analysis

To estimate the density of axon fibers, we converted the data of axon tracing from vector type to raster type using ADOBE PHOTOSHOP CS3 (Adobe Systems). After subdividing the GPe into 40 μm × 40 μm boxes, the density of GFP+ or RFP+ axon fibers in each box was determined using the histogram tool (ADOBE PHOTOSHOP CS3). The results were exported to Microsoft Excel 2013 software (Microsoft, Redmond, WA), and the heat map images were depicted in ADOBE ILLUSTRATOR CS3 software.

We modeled the density of axon fibers in a sample with two Gaussian distributions. The boxes were used as data points, each of which was weighted by its axonal density. Since the number of data points along the z-

axis (across slices) was much less than that along the x- and y-axes (within slices), we augmented data by copying each data point to 20 μm above and 20 μm below the slice containing it. For each sample, a total of 100 initial conditions were used to obtain the best fit of the model using mclust (Scrucca et al., 2016). The center of each Gaussian distribution on the rostral and caudal portions of the GPe was determined and named as Arborizations #1 and #2, respectively. The distances between two GFP+ and RFP+ arborizations (G1-G2 and R1-R2) and distances between overlapping GFP+ and RFP+ arborizations (G1-R1 and G2-R2) were compared by using the Kolmogorov-Smirnov test with R package software (version 3.4.1; R Development Core Team). We analyzed the distribution of axon fibers of dMSNs and iMSNs. After subdividing the GPe as described above, boxes containing GFP+ and/or RFP+ fibers were plotted in the parasagittal plane. The number of each box type was counted and then converted to the area. Finally, the proportion of boxes positive for GFP only, RFP only, or both GFP and RFP was calculated for all sections.

To examine the topographical projections, 3D coordinates of the infected cells were first determined by referring to the brain atlas (Franklin and Paxinos, 2007). Using the coordinates, the center of gravity of the infection site was calculated in each injection experiment. The centers of the infection sites and the centers of the arborizations were then plotted in the rostrocaudal, dorsoventral, and mediolateral planes. Linear regression analysis was performed using Microsoft Excel 2013 software.

The GPe was outlined in accordance with the cytoarchitecture, and CB-rich and CB-poor regions were distinguished as described above. The immunofluorescence intensities of GFP and RFP were measured using ImageJ software. The total intensities of GFP and RFP in the two regions were normalized to 1 arbitrary unit in each mouse, and the percentage of the intensities in each region was calculated. Multiple comparison tests were performed using one-way ANOVA followed by Tukey's *post hoc* test in R package software.

Supplemental References

Campbell, R.E., Tour, O., Palmer, A.E., Steinbach, P.A., Baird, G.S., Zacharias, D.A., and Tsien, R.Y. (2002). A monomeric red fluorescent protein. Proc Natl Acad Sci U S A 99, 7877-7882.

Franklin, K.B.J., and Paxinos, G. (2007). Paxinos and Franklin's The mouse brain in stereotaxic coordinates.

Furuta, T., Kaneko, T., and Deschenes, M. (2009). Septal neurons in barrel cortex derive their receptive field input from the lemniscal pathway. J Neurosci 29, 4089-4095.

Furuta, T., Tomioka, R., Taki, K., Nakamura, K., Tamamaki, N., and Kaneko, T. (2001). In vivo transduction of central neurons using recombinant Sindbis virus: Golgi-like labeling of dendrites and axons with membrane-targeted fluorescent proteins. J Histochem Cytochem 49, 1497-1508.

Gong, S., Doughty, M., Harbaugh, C.R., Cummins, A., Hatten, M.E., Heintz, N., and Gerfen, C.R. (2007). Targeting Cre recombinase to specific neuron populations with bacterial artificial chromosome constructs. J Neurosci 27, 9817-9823.

Hamamoto, M., Kiyokage, E., Sohn, J., Hioki, H., Harada, T., and Toida, K. (2017). Structural basis for cholinergic regulation of neural circuits in the mouse olfactory bulb. J Comp Neurol 525, 574-591.

Hioki, H., Kameda, H., Nakamura, H., Okunomiya, T., Ohira, K., Nakamura, K., Kuroda, M., Furuta, T., and Kaneko, T. (2007). Efficient gene transduction of neurons by lentivirus with enhanced neuron-specific promoters. Gene Ther 14, 872-882.

Hioki, H., Kuramoto, E., Konno, M., Kameda, H., Takahashi, Y., Nakano, T., Nakamura, K.C., and Kaneko, T. (2009). High-level transgene expression in neurons by lentivirus with Tet-Off system. Neurosci Res 63, 149-154.

Hioki, H., Nakamura, H., and Furuta, T. (2016). Application of Virus Vectors for Anterograde Tract-Tracing and Single-Neuron Labeling Studies. In Receptor and Ion Channel Detection in the Brain, R. Luján, and F. Ciruela, eds. (New York, NY: Humana Press), pp. 247-266.

Hioki, H., Nakamura, H., Ma, Y.F., Konno, M., Hayakawa, T., Nakamura, K.C., Fujiyama, F., and Kaneko, T. (2010). Vesicular glutamate transporter 3-expressing nonserotonergic projection neurons constitute a subregion in the rat midbrain raphe nuclei. J Comp Neurol 518, 668-686.

Kameda, H., Furuta, T., Matsuda, W., Ohira, K., Nakamura, K., Hioki, H., and Kaneko, T. (2008). Targeting green fluorescent protein to dendritic membrane in central neurons. Neurosci Res 61, 79-91.

Kataoka, N., Hioki, H., Kaneko, T., and Nakamura, K. (2014). Psychological stress activates a dorsomedial hypothalamus-medullary raphe circuit driving brown adipose tissue thermogenesis and hyperthermia. Cell Metab 20, 346-358.

Kuramoto, E., Furuta, T., Nakamura, K.C., Unzai, T., Hioki, H., and Kaneko, T. (2009). Two types of thalamocortical projections from the motor thalamic nuclei of the rat: a single neuron-tracing study using viral vectors. Cereb Cortex 19, 2065-2077.

Lee, T., Kaneko, T., Taki, K., and Mizuno, N. (1997). Preprodynorphin-, preproenkephalin-, and

preprotachykinin-expressing neurons in the rat neostriatum: an analysis by immunocytochemistry and retrograde tracing. J Comp Neurol 386, 229-244.

Moriyoshi, K., Richards, L.J., Akazawa, C., O'Leary, D.D., and Nakanishi, S. (1996). Labeling neural cells using adenoviral gene transfer of membrane-targeted GFP. Neuron 16, 255-260.

Nakamura, K.C., Kameda, H., Koshimizu, Y., Yanagawa, Y., and Kaneko, T. (2008). Production and histological application of affinity-purified antibodies to heat-denatured green fluorescent protein. J Histochem Cytochem 56, 647-657.

Okada, A., Lansford, R., Weimann, J.M., Fraser, S.E., and McConnell, S.K. (1999). Imaging cells in the developing nervous system with retrovirus expressing modified green fluorescent protein. Exp Neurol 156, 394-406.

Schnutgen, F., Doerflinger, N., Calleja, C., Wendling, O., Chambon, P., and Ghyselinck, N.B. (2003). A directional strategy for monitoring Cre-mediated recombination at the cellular level in the mouse. Nat Biotechnol 21, 562-565.

Scrucca, L., Fop, M., Murphy, T.B., and Raftery, A.E. (2016). mclust 5: Clustering, Classification and Density Estimation Using Gaussian Finite Mixture Models. R j 8, 289-317.

Sohn, J., Takahashi, M., Okamoto, S., Ishida, Y., Furuta, T., and Hioki, H. (2017). A Single Vector Platform for High-Level Gene Transduction of Central Neurons: Adeno-Associated Virus Vector Equipped with the Tet-Off System. PLoS One 12, e0169611.

Suzuki, Y., Kiyokage, E., Sohn, J., Hioki, H., and Toida, K. (2015). Structural basis for serotonergic regulation of neural circuits in the mouse olfactory bulb. J Comp Neurol 523, 262-280.

Tamamaki, N., Nakamura, K., Furuta, T., Asamoto, K., and Kaneko, T. (2000). Neurons in Golgi-stain-like images revealed by GFP-adenovirus infection in vivo. Neurosci Res 38, 231-236.
